# Screening of Onion (*Allium cepa* L.) Genotypes for Drought Tolerance Using Physiological and Yield Based Indices Through Multivariate Analysis

**DOI:** 10.3389/fpls.2021.600371

**Published:** 2021-02-09

**Authors:** Pranjali A. Gedam, A. Thangasamy, Dhananjay V. Shirsat, Sourav Ghosh, K. P. Bhagat, Onkar A. Sogam, A. J. Gupta, V. Mahajan, P. S. Soumia, Vanita N. Salunkhe, Yogesh P. Khade, Suresh J. Gawande, P. S. Hanjagi, R. Shiv Ramakrishnan, Major Singh

**Affiliations:** ^1^ICAR-Directorate of Onion and Garlic Research, Rajgurunagar, India; ^2^ICAR-National Institute of Abiotic Stress Management, Baramati, India; ^3^ICAR-National Rice Research Institute, Cuttack, India; ^4^Jawaharlal Nehru Krishi Vishwa Vidyalaya, Jabalpur, India

**Keywords:** onion, drought, genetic diversity, multivariate analysis, stress indices

## Abstract

Drought is a leading abiotic constraints for onion production globally. Breeding by using unique genetic resources for drought tolerance is a vital mitigation strategy. With a total of 100 onion genotypes were screened for drought tolerance using multivariate analysis. The experiment was conducted in a controlled rainout shelter for 2 years 2017–2018 and 2018–2019 in a randomized block design with three replications and two treatments (control and drought stress). The plant was exposed to drought stress during the bulb development stage (i.e., 50–75 days after transplanting). The genotypes were screened on the basis of the drought tolerance efficiency (DTE), percent bulb yield reduction, and results of multivariate analysis viz. hierarchical cluster analysis by Ward’s method, discriminate analysis and principal component analysis. The analysis of variance indicated significant differences among the tested genotypes and treatments for all the parameters studied, viz. phenotypic, physiological, biochemical, and yield attributes. Bulb yield was strongly positively correlated with membrane stability index (MSI), relative water content (RWC), total chlorophyll content, antioxidant enzyme activity, and leaf area under drought stress. The genotypes were categorized into five groups namely, highly tolerant, tolerant, intermediate, sensitive, and highly sensitive based on genetic distance. Under drought conditions, clusters II and IV contained highly tolerant and highly sensitive genotypes, respectively. Tolerant genotypes, viz. Acc. 1656, Acc. 1658, W-009, and W-085, had higher DTE (>90%), fewer yield losses (<20%), and performed superiorly for different traits under drought stress. Acc. 1627 and Acc. 1639 were found to be highly drought-sensitive genotypes, with more than 70% yield loss. In biplot, the tolerant genotypes (Acc. 1656, Acc. 1658, W-085, W-009, W-397, W-396, W-414, and W-448) were positively associated with bulb yield, DTE, RWC, MSI, leaf area, and antioxidant enzyme activity under drought stress. The study thus identified tolerant genotypes with favorable adaptive traits that may be useful in onion breeding program for drought tolerance.

## Introduction

Bulb onion (*Allium cepa* L.) is an important vegetable crops, with nearly 98 million tones produced globally (FAOSTAT, 2019). With a production of 23.2 million tones and export of 1.58 million tones, India is the second-largest producer of bulb onion next to China^1^. In India, onions are grown under diverse climatic conditions. A substantial part of the area under onion cultivation is dependent on monsoon rainfall for its water demand, and therefore, the onion crop is vulnerable to climatic abberations such as drought. Frequent drought episode that are linked with climate change have led to approximately 30% decrement in global bulb production. Depending on the growing season, the onion crop requires approximately 45 lakh liters hectare^–1^ water during its growth period which is quite high. Therefore, developing drought-tolerant onion varieties with promising adaptive traits is crucial for enhancing its productivity in water-scare regions.

Drought stress affects plant growth by altering various morphological, physiological, and metabolic processes (Diaz et al., 2010). It causes major damages to plants by disturbing water relations, inducing cellular membrane damage and forming of reactive oxygen species in plant tissues (Sairam and Saxena, 2000). Prolonged dry spell leads to poor plant growth and photosynthesis, which ultimately results in heavy yield losses. Phenotyping is a significant approach for screening germplasm based on morpho-physiological, biochemical, and yield performance (Passioura, 2012). Phenotypic attribute is the best criterion used for identifying tolerant genotypes among different crops based on their promising adaptive traits under drought stress (Vaezi et al., 2010; Mwadzingeni et al., 2016). In cereals, traits namely the plant height, number of productive tillers, spikelet per spike, and days to maturity are some of the important yield traits used for screening genotypes under limited water supply (Blum, 2010). Thus largely diverse phenotypic responses have been reported among different crop genotypes due to alteration in various physiological, biochemical, and molecular responses (Fenta et al., 2014; Ramakrishnan et al., 2016; Aghaie et al., 2018). This indicates variation in genotypic difference among the genotypes in terms of their drought tolerance level. Therefore from the breeding perspective, effective screening techniques should be used to identify tolerant genotypes that can perform better under limited water supply than the other genotypes.

Bulb yield in the onion crop has been reported to be directly associated with the amount of water supply. Information regarding genetic diversity among genotypes and the correlation among different traits under different water regimes is limited. Bulb yield is the primary trait that needs to be considered while evaluating drought tolerance along with secondary indicators, namely plant water status, and physiological, and biochemical parameters. The extent of damage to bulb yield depends on the genotype and phenological stage at which drought stress occurrs (Ghodke et al., 2018). Typically, genotypes with better drought-adaptive traits and relatively higher yield under water stress conditions need to be identified. Screening of genotypes based on few phenotypic traits and identifying the best criterion or trait among them is the major challenge while a screening large germplasm pool. Thus numerous genotypes can be more precisely evaluated at the same time by using appropriate statistical tools and employing multiple phenotypic, physiological, biochemical, and yield traits.

Multivariate cluster analysis and principal component analysis (PCA) are reliable tools for grouping genotypes based on their performance under water stress. These tools also characterize the genetic divergence among the tested genotypes. Thus, selecting appropriate drought-tolerant onion genotypes from the available germplasm collection is a prime concern for developing improved varieties and hybrids. Thus, the present study screened and evaluated the response of 100 onion genotypes to drought stress at the bulb development stage. The identified tolerant genotype with drought-adaptive traits can significantly improve onion productivity in low rainfall areas and advance our understanding of the plant response to drought stress at physiological and biochemical levels.

## Materials and Methods

### Plant Material and Experimental Site

A total of 100 onion genotypes were used as the plant material in the present study. All these genotypes were selected from the germplasm collection available at ICAR-Directorate of Onion and Garlic Research (ICAR-DOGR), Pune, India (N 18°84′, E 73°88′, and 553.8 m above mean sea level). The controlled experiment was conducted in an automated rain-out shelter at ICAR-DOGR following randomized block design after monsoon season (Supplementary Figure 1) from, November to April for two successive years (2017–2018 and 2018–2019). The soil of experimental plots was sandy loam in texture with 32–35% clay, 40% sand, and 20% silt with 7.9 pH. The recommended dose of potassium, phosphorus, and sulfur, and half dose of nitrogen were applied at the time of transplanting and remaining nitrogen was applied in splits doses at 30, 45, and 60 days after transplanting (DAT). At all stages of crop growth, recommended plant protection procedures and hand weeding were performed to combat weeds, pests, and disease.

### Drought Treatment

Initially the seedlings for each genotypes were grown in the nursery on raised beds. Seeds were coated with fungicide (Thiram 1 g kg^–1^) before sowing in the nursery to avoid fungal and soil-borne diseases. Six week healthy seedlings were transplanted in the rain-out shelter on raised bed of 1.2 m in width and 30 cm in height by maintaining a spacing of 15 cm row to row and 10 cm plant to plant in three replicates comprising of 15 seedlings in each replication. Regular water supply was maintained in both the plots (control and drought) until 50 DAT. To evaluate drought tolerance, two water regimes were maintained, namely well-watered condition (control with 90% field capacity) and water-deficit stress (50% field capacity). For this, water supply was stopped in the drought plots for continuous 25 days during the bulb enlargement stage (50–75 DAT), whereas the plants in the control plots were maintained uninterruptedly hydrated as per their requirement. Gravimetric soil moisture content was measured by randomly collecting the soil sample from a depth of 55–60 cm on every alternate day during the stress period. The automated rain-out shelter protected the experiment from any possible seasonal rainfall. Data on all phenotypic and physiological traits were colleted from both the plots, and leaf samples were collected in liquid nitrogen and stored for biochemical analysis.

### Morphological Traits

Morphological traits such as plant height, leaves per plant, and leaf area of well-watered and water-stressed plants were recorded on 50 and 75 DAT. Plant height was determined by measuring from the base to the tip of the longest leaves. The total number of leaves per plant was monitored after stress treatment. The leaf area of each genotype from both the water regimes was measured using a LICOR 3100 leaf area meter. All observations were recorded in replicates for each studied genotypes. The means of all the recorded parameters for 2 years were pooled (physiological, biochemical, and yield) and analyzed for screening, and evaluating genotypic performance under well-watered and water-deficit stress conditions.

### Physiological Traits

Relative water content (RWC), membrane stability index (MSI), and total chlorophyll content were measured for each genotype and their means for 2 years were pooled from both the water regimes after relieving stress.

#### Relative Water Content

Leaf RWC was measured from a fully grown fourth leaf of each genotype. A fresh leaf sample of 0.5 g fresh weight (FW) was kept in distilled water for 4 h to record its turgid weight (TW). Turgid leaf samples were then kept for drying in a hot air oven at 70°C until the constant dry weight (DW) was attained. RWC was calculated using the formula given by Barrs and Weatherley (1962):

$$R\,W\,C=\frac{\left(F\,W-D\,W\right)}{\left(T\,W-D\,W\right)}\times 100$$

#### Membrane Stability Index

Drought-induced cellular membrane injury was monitored by measuring the MSI according to the procedure of Sairam et al. (1997). Leaves from both the treatments were collected, cut into small 2 cm discs, and washed with distilled water to remove debris if any from the leaf surface. MSI was estimated by taking 100 mg of the leaf disc in two test tubes each containing 10 mL of double-distilled water. The first test tube was heated at 40°C in a water bath for 30 min, and the electrical conductivity was measured on a conductivity bridge (C1). The second test tube was kept at 100°C for 10 min in a boiling water bath to record the conductivity on the conductivity bridge (C2). MSI was calculated in percentage by using the following formula as:

$$M\,S\,I=\left[1-\frac{C\,1}{C\,2}\right]\times 100.$$

#### Total Chlorophyll

By using the non-maceration method (Hiscox and Israelstam, 1979), Total Chlorophyll was measured spectrophotometrically (UV-Visible Spectrophotometer, Thermo, United States) by incubating a leaf sample (0.05 g) in the test tubes containing 10 mL of dimethyl sulfoxide. The test tubes were placed in a water bath maintained at 60°C for 60 min. Absorbance was recorded at 645 and 663 nm after placing the tubes at room temperature for 30 min. Total chlorophyll was calculated using the following formula given by Arnon (1949):

$$\mathrm{Total}\,\mathrm{Chlorophyll}=\frac{\begin{array}{c} \left(20.2\times O\,D_{645}+8.02\times O\,D_{663}\right)\times \mathrm{Voleme}\,\mathrm{of}\,\mathrm{extract}\\ \times \mathrm{Weight}\,\mathrm{of}\,\mathrm{sample} \end{array}}{\begin{array}{c} 1000 \end{array}}$$

Where; OD_663_ is absorbance at 663 nm and OD_645_ is absorbance at 645 nm.

### Biochemical Traits

Various biochemical traits namely phenol content, pyruvic acid, antioxidant enzyme activity, and total soluble solids (TSS) content were measured for each genotype and their means for years were pooled from both the water regimes after relieving stress.

#### Total Phenolic Content

The total phenolic content of the leaf sample was determined using the Folin-Ciocalteau (FC) reagent according to the method of Singleton and Rossi (1965) using gallic acid as the standard. The fresh leaf sample (1 g) was homogenized in 10 mL of 80% aqueous methanol and the homogenate was centrifuged at 5000 rpm for 10 min. A mixture of 200 μL supernatant and 1 mL FC reagent was incubated at room temperature for 5 min. After incubation, 800 μL of sodium carbonate was added to the reaction mixture and further incubated in dark at room temperature for 2 h. The absorbance was recorded at 765 nm. Phenol content was expressed as mg gallic acid equivalent g^–1^ dry weight.

#### Pyruvic Acid

Pyruvic acid was estimated spectrophotometrically through the method of Schwimmer and Weston (1961) using sodium pyruvate as the standard. The core of the bulb sample (1 g) was homogenized in 1 mL double distilled water and squeezed in a muslin cloth and allowed to settle at room temperature for 10 min. Then 0.5 mL of extract and 1.5 mL of 5% trichloroacetic acid were vortexed and diluted with 20 mL with double distilled water. The reaction mixture containing 1 mL extract, 1 mL double distilled water, and 1 mL dinitrophenylhydrazine reagent was incubated at 37°C in a water bath for 10 min and 5 mL of 0.6 M sodium hydroxide was added to this mixture. Absorbance was then recorded at 420 nm. Pyruvic acid was expressed as the μmole g^–1^ sample.

#### Antioxidant Enzyme Activity

The total antioxidant enzyme activity was determined spectrophotometrically through Ferric Ion Reducing Antioxidant Power (FRAP) assay as described by Stratil et al. (2006) by using ascorbic acid as the standard. The FRAP reagent was prepared freshly with 25 mL of 300 mM sodium acetate buffer, 2.5 mL of 10 mM 2,4,6-Tripyridile-S-Triazine, and 2.5 mL of 20 mM FeCl_3_ prepared in 40 mM HCl and incubated at 37°C in a water bath for 10 min. An aliquot of 150 μL of the leaf extract was prepared in 80% aqueous methanol, mixed with 2850 μL of the FRAP reagent, and incubated in dark at room temperature for 30 min. The absorbance of the ferrous tripyridyltriazine complex was recorded at 593 nm against methanol as the blank. The FRAP value was expressed in μg g^–1^ dry weight of sample.

#### Total Soluble Solids

The TSS content of the bulb sample immediately after harvest was measured using a digital refractometer and the values are presented in Brix.

### Bulb Yield and Drought Tolerance Indices

After more than 70% of the neck fall occurred at the end of the season, the bulbs were harvested separately for each genotype. Bulb yield was recorded separately from both the water regimes for each tested genotype, and their means for 2 years were pooled for further analysis. Bulbs were assessed visually to detect any sprouting or rotting losses caused by deficit irrigation. Drought tolerance indices based on bulb yield under the two water regimes were calculated as follows

$$•\mathrm{Drought}\,\mathrm{tolerance}\,\mathrm{efficiency}\,\left(\text{DTE}\%\right)=\frac{\mathrm{Yield}\,\mathrm{under}\,\mathrm{drought}\,\mathrm{stress}}{\mathrm{Yield}\,\mathrm{under}\,\mathrm{well}\,\mathrm{watered}}\times 100;$$

(Fischer and Wood, 1981)

$$•\mathrm{Stress}\,\mathrm{tolerance}\,\mathrm{index}\,\left(\text{STI}\right)=\frac{\left(\mathrm{Yield}\,\mathrm{under}\,\mathrm{drought}\,\mathrm{stress}\times \mathrm{Yield}\,\mathrm{under}\,\mathrm{well}-\mathrm{watered}\right)}{{\left(\mathrm{Mean}\,\mathrm{yield}\,\mathrm{of}\,\mathrm{all}\,\mathrm{genotypes}\,\mathrm{under}\,\mathrm{well}-\mathrm{watered}\right)}^{2}};$$

(Fernandez, 1992)

$$•\mathrm{Drought}\,\mathrm{susceptibility}\,\mathrm{index}\,\left(\text{DSI}\right)=\frac{1-\mathrm{Yield}\,\mathrm{under}\,\mathrm{drought}\,\mathrm{stress}\,/\,\mathrm{Yield}\,\mathrm{under}\,\mathrm{well}-\mathrm{watered}}{\mathrm{Stress}\,\mathrm{Intensity}}$$

Where,

$$\mathrm{Stress}\,\mathrm{Intensity}\;=\frac{1-\mathrm{Mean}\,\mathrm{yield}\,\mathrm{of}\,\mathrm{all}\,\mathrm{genotypes}\,\mathrm{under}\,\mathrm{drought}}{\mathrm{Mean}\,\mathrm{yield}\,\mathrm{of}\,\mathrm{all}\,\mathrm{genotypes}\,\mathrm{under}\,\mathrm{well}\,\mathrm{watered}};$$

(Fischer and Maurer, 1978)

$$•\mathrm{Mean}\,\mathrm{relative}\,\mathrm{performance}\,\left(\text{MRP}\right)=\frac{\mathrm{Yield}\,\mathrm{under}\,\mathrm{stress}}{\mathrm{Mean}\,\mathrm{yield}\,\mathrm{of}\,\mathrm{all}\,\mathrm{genotypes}\,\mathrm{under}\,\mathrm{stress}}+\frac{\mathrm{Yield}\,\mathrm{under}\,\mathrm{well}\,\mathrm{watered}}{\mathrm{Mean}\,\mathrm{yield}\,\mathrm{of}\,\mathrm{all}\,\mathrm{genotypes}\,\mathrm{under}\,\mathrm{well}\,\mathrm{watered}};$$

(Raman et al., 2012)

$$•\mathrm{Mean}\,\mathrm{productivity}\,\left(\text{MP}\right)=\frac{\mathrm{Yield}\,\mathrm{under}\,\mathrm{stress}+\mathrm{Yield}\,\mathrm{under}\,\mathrm{well}\,\mathrm{watered}}{2};$$

(Raman et al., 2012).

### Analysis of Genetic Parameters

The data was subjected to analysis, the genotypic and phenotypic correlations were calculated by the technique given by Kwon and Torrie (1964) by following formula,

Genetic Variance (Vg) = Genotypic Mean Square (GMS)−Error Mean Square (EMS)/Number of Replications (r)

E⁢n⁢v⁢i⁢r⁢o⁢n⁢m⁢e⁢n⁢t⁢a⁢l⁢⁢V⁢a⁢r⁢i⁢a⁢n⁢c⁢e=E⁢r⁢r⁢o⁢r⁢⁢m⁢e⁢a⁢n⁢⁢S⁢q⁢u⁢a⁢r⁢e

P⁢h⁢e⁢n⁢o⁢t⁢y⁢p⁢e⁢⁢v⁢a⁢r⁢i⁢a⁢n⁢c⁢e⁢⁢(V⁢p)=V⁢g+V⁢e⁢/⁢r

The genotypic coefficient of variation (GCV) and phenotypic coefficient of variation (PCV) were calculated as per following formula,

Genetic coefficient of variation % = VgX×100

Phenotypic coefficient of variation % = VpX×100

The heritability (H^2^) on entry mean basis was calculated by *t* formula,

$$\mathrm{Heritability}=\frac{V\,p}{V\,g}$$

### Statistical Analysis

Physiological, biochemical, and yield data for 2 years were analyzed using SAS software (Version 9.3; SAS Institute, Cary, NC, United States). Combined analysis of variance (ANOVA) and the least significance test were performed using SAS software to test the genotypic difference, drought stress effect and to compare the phenotypic value of the genotype for specific traits under both water regimes. The mean data (pooled) for the recorded traits for 2 years were used for calculating Pearson’s correlation coefficient and the association between different traits under well-watered and water-deficit stress conditions by using SPSS software (Version 16.0). PCA and biplot PCA were performed for genotypes and traits under water-deficit stress by using SPSS and XLSTAT software to show the relationships among the tested genotypes based on different traits.

## Results

### Effect of Drought Stress on Stress Indices

Eleven physiological traits and two stress indices were selected for screening 100 onion genotypes for their drought tolerance. The genotypes were classified into five groups according to 2 years pooled data of percent bulb yield reduction (Table 1). Yield decreased gradually in all genotypes exposed to drought stress during the bulb enlargement stage (50–75 DAT) irrespective of their year of planting. Eleven genotypes with less than 20% reduction in bulb yield were identified as highly tolerant to drought, whereas, 22 genotypes with 20–40% yield reduction as tolerant to drought. Twenty-three genotypes with 40–60% yield reduction were categorized as an intermediate group. Fourteen genotypes with yield reduction of more than 70% and poor plant stand were classified as a highly sensitive group whereas, 30 genotypes with a 60–70% yield reduction as a sensitive group. Another reliable stress index used to screen the genotypes under water stress was the drought tolerance efficiency (DTE). In total, 11 genotypes were identified with more than 80% DTE and hence categorized as highly tolerant to drought whereas, 22 genotypes with 60–80% DTE were grouped as tolerant. Likewise, 23 genotypes with 40–60% DTE were identified as an intermediate group. Fourteen genotypes recorded with less than 30% DTE were categorized as highly sensitive to drought whereas 30 genotypes with 30–40% DTE were grouped as sensitive genotypes (Table 2). Thus, based on DTE and bulb yield reduction, the genotypes were classified into different groups. Acc. 1656, Acc. 1658, W-009, and W-085 with more than 90% DTE and less than 20% yield reduction were identified as highly drought-tolerant genotypes. Two entries (Acc. 1627 and Acc. 1639) and three varieties (Bhima Raj, Bhima Light Red, and Phule Safed) with less than 20%, DTE and more than 80% yield reduction were identified as highly drought-sensitive genotypes. Thus, DTE and bulb yield can act as promising indicators of genotypes with improved drought tolerance.

**TABLE 1:** Genotypes grouped based on yield loss due to drought stress.

Genotypes	Yield reduction (<20%)	Genotypes	Yield reduction (20–40%)	Genotypes	Yield reduction (40–60%)	Genotypes	Yield reduction (60–70%)	Genotypes	Yield reduction (>70%)
W 085	6.65	W 453	20.39	Acc. 1626	41.16	RGP-4	60.00	Acc. 1652	70.89
Acc. 1656	7.05	N-2-4-1	20.81	W 440	42.12	W 344	60.05	KH-M-4	74.41
W 009	7.73	W 419	20.89	Bhima Shweta	42.29	DOGR Hybrid-50	60.88	Acc. 1629	75.27
Acc. 1658	9.52	Acc. 1663	21.11	MS 100 × Bhima Shweta	43.08	DOGR Hybrid-5	61.14	KH-M-3	76.08
W 448	10.12	Acc. 1613	21.18	Bhima Shubhra	43.32	W 355	62.35	Acc. 1651	76.95
W 397	13.27	Acc. 1635	21.92	DOGR 1047	44.08	Acc. 1633	62.43	Acc. 1615	77.04
W 444	15.25	DOGR 1048	22.57	MS 100 × W-448	45.32	RGP-3	62.67	Acc. 1655	77.17
Acc. 1608	16.98	W 340	22.87	Acc. 1666	45.36	DOGR Hybrid-441	62.81	Acc. 1661	78.30
W 414	18.34	W 441	23.03	W 361	47.72	Bhima Red	63.00	Acc. 1668	80.04
Acc. 1622	18.72	Acc. 1649	23.31	MS 100 × W-361	49.76	Acc. 1669	63.06	Acc. 1627	83.11
W 439	19.29	W 396	23.35	DOGR Hybrid-1	51.95	DOGR Hybrid-6	63.53	Acc. 1639	84.05
		RGP-2	25.18	KH-M-2	53.96	Acc. 1664	63.76	Phule Safed	84.12
		Acc. 1609	25.69	DOGR 1050	54.51	KH-M-1	64.05	Bhima Light Red	84.29
		Acc. 1657	25.95	Acc. 1623	55.97	Acc. 1621	64.17	Bhima Raj	85.59
		DOGR 1044	27.30	Acc. 1632	56.66	Acc. 1700	64.41		
		W 408	35.27	Acc. 1612	56.75	Acc. 1694	64.94		
		Acc. 1630	35.33	Acc. 1617	56.91	Acc. 1660	65.20		
		W 043	35.65	DOGR 1172	57.08	P-1-3	65.46		
		W 394	36.24	DOGR Hybrid-7	57.69	Acc. 1637	66.29		
		DOGR Hybrid-8	37.91	Acc. 1636	58.04	W 132	66.76		
		546 DR	37.97	Acc. 1625	58.20	Bhima Safed	66.77		
		Bhima Kiran	38.08	DOGR Hybrid-2	58.22	Bhima Super	67.04		
				RGP-5	58.50	Bhima Shakti	67.57		
						W 306	67.72		
						Acc. 1646	67.80		
						Acc. 1619	68.30		
						W 172	68.52		
						RGP-1	68.53		
						Bhima Dark Red	68.90		
						W 504	69.46		

**TABLE 2:** Genotypes grouped based on drought tolerance efficiency (DTE %).

Genotypes	DTE (<30%)	Genotypes	DTE (30–40%)	Genotypes	DTE (40–60%)	Genotypes	DTE (60–80%)	Genotypes	DTE (>80%)
Bhima Raj	14.41	W 504	30.54	RGP-5	41.50	Bhima Kiran	61.92	W 439	80.71
Bhima Light Red	15.71	Bhima Dark Red	31.10	DOGR Hybrid-2	41.78	546 DR	62.03	Acc. 1622	81.28
Phule Safed	15.88	RGP-1	31.47	Acc. 1625	41.80	DOGR Hybrid-8	62.09	W 414	81.66
Acc. 1639	15.95	W 172	31.48	Acc. 1636	41.96	W 394	63.76	Acc. 1608	83.02
Acc. 1627	16.89	Acc. 1619	31.70	DOGR Hybrid-7	42.31	W 043	64.35	W 444	84.75
Acc. 1668	19.96	Acc. 1646	32.20	DOGR 1172	42.92	Acc. 1630	64.67	W 397	86.73
Acc. 1661	21.70	W 306	32.28	Acc. 1617	43.09	W 408	64.73	W 448	89.88
Acc. 1655	22.83	Bhima Shakti	32.43	Acc. 1612	43.25	DOGR 1044	72.70	Acc. 1658	90.48
Acc. 1615	22.96	Bhima Super	32.96	Acc. 1632	43.34	Acc. 1657	74.05	W 009	92.27
Acc. 1651	23.05	Bhima Safed	33.23	Acc. 1623	44.03	Acc. 1609	74.31	Acc. 1656	92.95
KH-M-3	23.92	W 132	33.24	DOGR 1050	45.49	RGP-2	74.82	W 085	93.35
Acc. 1629	24.73	Acc. 1637	33.71	KH-M-2	46.04	W 396	76.65		
KH-M-4	25.59	P-1-3	34.54	DOGR Hybrid-1	48.05	Acc. 1649	76.69		
Acc. 1652	29.11	Acc. 1660	34.80	MS 100 × W-361	50.24	W 441	76.97		
		Acc. 1694	35.06	W 361	52.28	W 340	77.13		
		Acc. 1700	35.59	Acc. 1666	54.64	DOGR 1048	77.43		
		Acc. 1621	35.83	MS 100 × W-448	54.68	Acc. 1635	78.08		
		KH-M-1	35.95	DOGR 1047	55.92	Acc. 1613	78.82		
		Acc. 1664	36.24	Bhima Shubhra	56.68	Acc. 1663	78.89		
		DOGR Hybrid-6	36.47	MS 100 × Bhima Shweta	56.92	W 419	79.11		
		Acc. 1669	36.94	Bhima Shweta	57.71	N-2-4-1	79.19		
		Bhima Red	37.00	W 440	57.88	W 453	79.61		
		DOGR Hybrid-441	37.19	Acc. 1626	58.84				
		RGP-3	37.33						
		Acc. 1633	37.57						
		W 355	37.65						
		DOGR Hybrid-5	38.86						
		DOGR Hybrid-50	39.12						
		W 344	39.95						
		RGP-4	40.00						

### Analysis of Variance

The combined ANOVA evaluated under well-watered and water-deficit stress conditions showed highly significant (*p* ≤ 0.05) genotypic differences in the physiological parameters (plant height, leaves per plant, leaf area, RWC, MSI, and chlorophyll), biochemical traits (phenol, antioxidant enzyme activity, pyruvic acid, and TSS), yield components and all possible relationship among them (Table 3). The findings indicated the extent of differences among the genotypes that would be helpful in selecting promising genotypes with drought tolerance. Genotypes into environment interactions were also significant for the studied parameters, indicating that most genotypes performed contrastingly under different water regimes. Thus the significant variance among the genotypes, environment, and genotypes × environment indicated that the studied traits were greatly influenced by both genotypes and water stress conditions.

**TABLE 3:** Combined ANOVA (mean square) for physiological, biochemical and drought stress indices under well-watered and stress condition.

Source	DF	MSI	Chl.	RWC	YLD	NL	LA	PH	PA	PHE	AOX	TSS
Environment	1	5460485**	41140.66**	162259**	23692.62**	984.04**	4067.78**	873.13**	28252.1**	253.08**	2096.11**	1637.64**
Rep (Env.)	4	7.54*	0.9922	2.86*	2.19	6.18*	1.39	2.15*	2.24	10.08*	2.71*	13.02*
Genotype	99	167.95**	75.41**	232.78**	141.72**	2.94**	31.33**	33.47**	995.13**	78.06**	842.63**	76.9**
Env.*Geno	99	160.88**	61.14**	224.48**	132.03**	2.43**	19.83**	8.4**	143.08**	46.64**	138.32**	8.41**
Error	396											
Total	599											

MSI, membrane stability index; Chl, chlorophyll; RWC, relative water content; YLD, bulb yield; NL, leaves Plant^–1^; LA, leaf area; PH, plant height; PA, pyruvic acid; PHE, total phenols; AOX, antioxidant enzyme activity; TSS, total soluble solids; DF, degree of freedom.

*, ** significant at 5 and 1% level of significance, respectively.

### Association of Parameters Under Drought Stress

Correlation analysis was performed among the physiological, biochemical, and yield contributing traits of 100 genotypes under well-watered and water-deficit stress conditions, and Pearson’s correlation matrix is presented in Table 4. Under drought stress conditions, a significant and strong positive correlation was observed between bulb yield and RWC (0.766^∗∗^), chlorophyll (0.591^∗∗^), MSI (0.642^∗∗^), and antioxidant enzyme activity (0.347^∗∗^) whereas the number of leaves (−0.298^∗^) negatively correlated with bulb yield. Likewise, chlorophyll was significantly and positively associated with MSI (0.542^∗∗^), RWC (0.697^∗∗^), TSS (0.405^∗∗^), antioxidant enzyme activity (0.247^∗^), and phenol (0.496^∗∗^) but negatively correlated with the number of leaves (−0.256^∗^) under water stress. The promising phenotypic trait that is, leaf area, was positively associated with MSI (0.299^∗^), RWC (0.200^∗^), antioxidant enzyme activity (0.212^∗^), and pyruvic acid (0.292^∗^), whereas RWC with MSI (0.755^∗∗^), antioxidant enzyme activity (0.392^∗∗^), phenol (0.651^∗∗^), TSS (0.230^∗^), and pyruvic acid (0.244^∗^) under limited water supply. Further, a significantly stronger association was observed between phenols and pyruvic acid (0.410^∗∗^) under water stress. Under the well-watered condition, the yield was positively associated with leaf area (0.206^∗^), whereas, pyruvic acid with phenols (0.386^∗∗^) and antioxidant enzyme activity (0.303^∗^). Chlorophyll content was negatively associated with pyruvic acid (−0.266^∗^), antioxidant enzymes activity (−0.349^∗^), and MSI (−0.236^∗^), but positively correlated with TSS (0.366^∗^). The drought stress indicator MSI was positively associated with antioxidant enzyme activity (0.302^∗^) in the control group. Thus, the correlation matrix under water-deficit stress signifies the association of various physiological and biochemical traits with bulb yield. The correlation matrix among different yield indices showed a strong positive association among stress tolerance index (STI), DTE, Mean productivity (MP), and mean relative performance (MRP), whereas a negative association of these indices was observed with stress susceptibility index (SSI) (−0.757^∗∗^) and bulb yield reduction (−0.924^∗∗^) under water stress (Table 5). Further, as the water stress increased, SSI was strongly and positively associated with bulb yield reduction (0.853^∗∗^). Taken together, strong negative and positive association was found between various parameters under water-deficit stress and these associations can be employed for identifying promising drought-responsive traits.

**TABLE 4:** Pearson’s correlation matrix among the physiological and yield traits under well-water (Upper diagonal) and water-deficit stress condition (Lower diagonal).

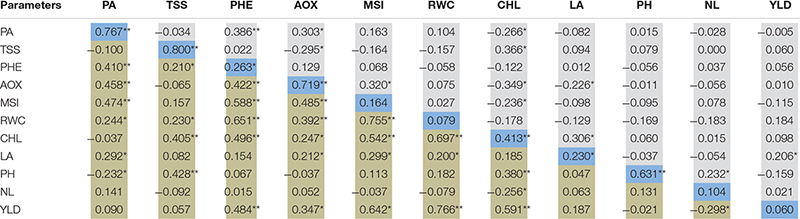

PA, pyruvic acid; TSS, total soluble solids; PHE, total phenols; AOX, antioxidant enzyme activity; MSI, membrane stability index; RWC, relative water content; CHL, chlorophyll; LA, leaf area; PH, plant height; NL, leaves Plant^–1^; YLD, bulb yield.

*, ** significant at 5 and 1% level of significance, respectively.

**TABLE 5:** Pearson’s correlation matrix among yield indices under drought stress.

Parameters	STI	DTE	MP	MRP	SSI	YL%	GMP	HMP
STI	1	0.924**	0.973**	0.676**	−0.757**	−0.924**	0.910**	0.986**
DTE		1	0.830**	0.367*	−0.853**	−1.000**	0.994**	0.954**
MP			1	0.822**	−0.630**	−0.830**	0.807**	0.936**
MRP				1	–0.168	−0.367*	0.331*	0.583**
SSI					1	0.853**	−0.904**	−0.847**
YL%						1	−0.994**	−0.954**
GMP							1	0.956**
HMP								1

STI, stress tolerance index; YSI, yield susceptibility index; DTE, drought tolerance efficiency; MP, mean productivity; MRP, mean relative performance; SSI, stress susceptibility index; YL%, yield loss percentage; GMP, geometric mean productivity; HMP, harmonic mean productivity.

*, ** significant at 5 and 1% level of significance, respectively.

### Descriptive Statistics

Descriptive statistics classified the 100 genotypes into five clusters using Ward’s method of clustering, based on square Euclidean distance between different traits as presented in Table 6. Compared with the other three clusters, the mean value for important physiological parameters was higher in cluster II followed by cluster I. Genotypes of cluster II recorded significantly higher mean values for DTE (81.21), chlorophyll (5.96), MSI (73.86), RWC (71.99), antioxidant enzyme activity (3.16), and yield (31.07) than other genotypes in different clusters. The mean value of significant traits from cluster IV and cluster V was lower than that of respective parameters of other genotypes. Divergence analysis revealed that among the studied traits, DTE and percent yield reduction contributed maximum (15%) toward genetic divergence of the studied genotypes, followed by bulb yield (14.6%), RWC (13%), and MSI (11%) (Table 7), whereas, TSS contributed the least (0.7%). Thus, these traits can be exploited for identifying genetically diverse parents for a breeding program. The PCV, GCV, broad-sense heritability (h^2^), and genetic advancement are presented as a percent of the mean for 12 traits. The value for the PCV was higher than that for the GCV for all the studied traits. The maximum estimated value for the PCV and the GCV was for DTE (479.98 and 477.97, respectively), followed by yield reduction (71.84 and 71.48, respectively), whereas, the minimum estimated value was for leaves per plant (0.38 and 0.12, respectively) and TSS (0.72 and 0.71, respectively). Heritability is the degree of influence of genotype and environment on the expression of the parameters. In our study, the estimated heritability values did not vary significantly among the traits except for leaves per plant (0.32), for the studied genotypes (Table 8).

**TABLE 6:** Descriptive statistics for all traits of 100 onion genotypes under different clusters.

Parameters	Cluster I		Cluster II		Cluster III		Cluster IV		Cluster V		Total	
						
	Mean	SD	Mean	SD	Mean	SD	Mean	SD	Mean	SD	Mean	SD
DTE	59.32	4.06	81.21	6.16	39.00	4.73	20.27	3.98	32.58	2.85	46.48	4.35
SSI	0.70	0.12	0.24	0.09	1.64	0.33	4.37	1.28	2.13	0.20	1.82	0.40
YL%	40.68	4.06	18.79	6.16	61.00	4.73	79.73	3.98	67.42	2.85	53.52	4.35
TSS	13.84	0.69	13.14	0.85	13.41	0.94	12.96	0.40	12.61	0.65	13.19	0.70
AOX	3.18	1.28	3.16	0.95	2.95	1.11	1.68	0.46	1.79	0.77	2.55	0.91
MSI	72.15	1.44	73.86	1.92	72.17	1.81	66.22	1.41	65.95	1.63	70.07	1.64
RWC	65.12	3.47	71.99	3.75	65.20	2.52	56.81	2.44	57.70	1.75	63.36	2.79
CHL	5.68	1.24	5.96	1.16	4.78	1.07	2.70	1.08	3.03	1.23	4.43	1.16
LA	35.76	2.57	34.99	3.59	35.93	2.83	32.14	4.23	31.44	4.78	34.05	3.60
PH	46.17	6.17	44.65	5.87	47.68	7.06	42.99	4.23	41.37	4.56	44.57	5.58
NL	6.51	0.71	6.30	0.54	6.85	0.60	6.47	0.43	6.80	0.62	6.59	0.58
YLD	22.18	3.06	31.07	2.92	14.33	1.92	8.31	1.67	12.63	1.29	17.70	2.17
	Tolerant		Highly Tolerant		Intermediate		Highly Susceptible		Susceptible			

*DTE, drought tolerance efficiency; SSI, stress susceptibility index; YL%, yield loss percentage; TSS, total soluble solids; AOX, antioxidant enzyme activity; MSI, membrane stability index; RWC, relative water content; CHL, chlorophyll; LA, leaf area; PH, plant height; NL, leaves plant^–1^; YLD, bulb yield.*

**TABLE 7:** Contribution of different physiological, biochemical and yield traits toward genetic divergence in 100 onion genotypes.

Parameters	Contribution (%)
DTE	15.230
SSI	13.666
YL%	15.230
TSS	0.701
AOX	3.800
MSI	11.027
RWC	13.094
CHL	9.730
LA	1.508
PH	0.245
NL	1.108
YLD	14.659

*DTE, drought tolerance efficiency; SSI, stress susceptibility index; YL%, yield loss percentage; TSS, total soluble solids; AOX, antioxidant enzyme activity; MSI, membrane stability index; RWC, relative water content; CHL, chlorophyll; LA, leaf area; PH, plant height; NL, leaves plant^–1^; YLD, bulb yield.*

**TABLE 8:** Estimates of genetic parameters for 12 quantitative traits of 100 onion genotypes under drought stress condition.

Parameters	GCV	PCV	h^2^ (Broad sense)	Genetic Advancement 5%	Genetic advancement as % of mean 5%
DTE	477.97	479.98	0.99	44.94	89.5
SSI	1.84	1.88	0.97	2.76	181.59
YL%	477.97	479.98	0.99	44.94	90.27
TSS	0.71	0.72	0.97	1.71	12.93
AOX	1.3	1.3	0.99	2.35	84.84
MSI	10.93	10.96	0.99	6.8	9.54
RWC	34.96	35.03	0.99	12.16	18.66
CHL	2.56	2.58	0.99	3.28	68.44
LA	13.06	13.69	0.95	7.27	20.92
PH	38.83	40.27	0.96	12.6	27.72
NL	0.123	0.38	0.32	0.41	6.22
YLD	71.48	71.84	0.99	17.37	91.43

*DTE, drought tolerance efficiency; SSI, stress susceptibility index; YL%, yield loss percentage; TSS, total soluble solids; AOX, antioxidant enzyme activity; MSI, membrane stability index; RWC, relative water content; CHL, chlorophyll; LA, leaf area; PH, plant height; NL, leaves plant^–1^; YLD, bulb yield; GCV, genetic coefficient of variance; PCV, phenotypic coefficient of variance; h^2^, heritability.*

### Cluster Analysis

**FIGURE 1:**
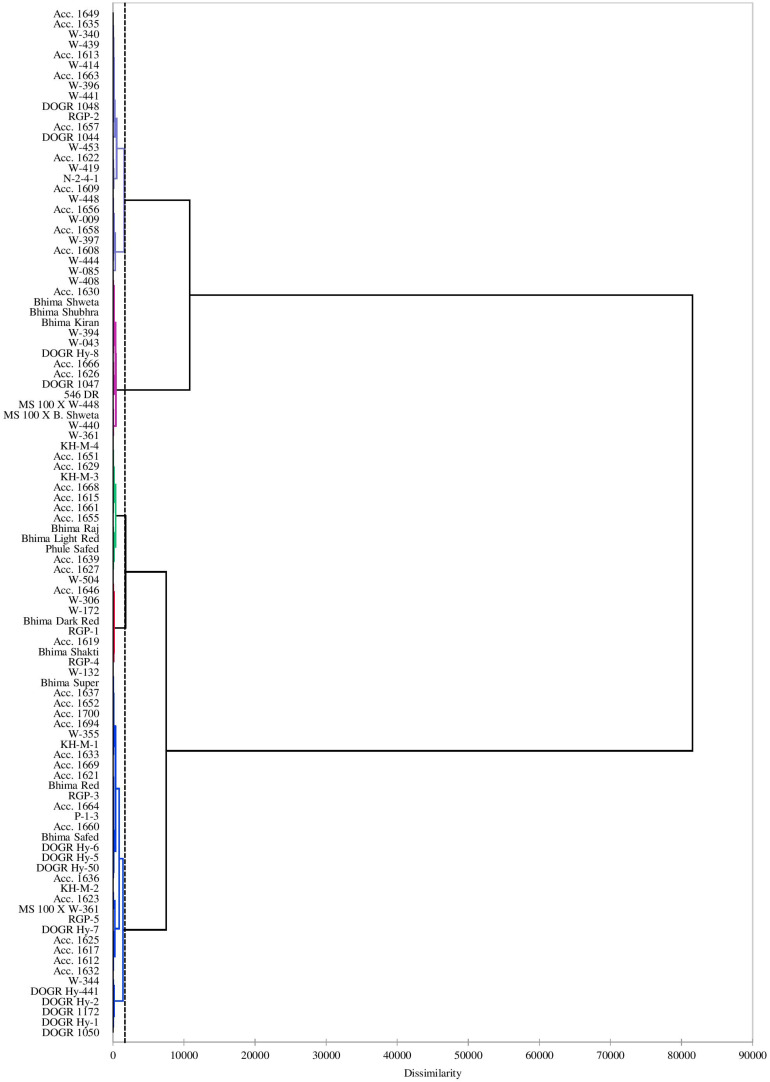
Dendrogram using Ward’s method presenting distribution of 100 onion genotypes under drought stress.

Cluster analysis performed using a dendrogram grouped the genotypes into five clusters based on the studied traits indicating the presence of greater genetic diversity among the genotypes in different clusters as represented by the rescaled Euclidean distance in Figure 1. The corresponding genotypes in each cluster are shown in Table 9 and their characteristics are described as below:

**TABLE 9:** Grouping of 100 onion genotypes into six clusters based on Euclidean analysis.

Cluster group	No of genotypes	Name of genotypes
Cluster I	16	546 DR, Acc. 1626, Acc. 1630, Acc. 1666, W-043, W-361, W-394, W-408, W-440, DOGR 1047, DOGR Hybrid-8, MS 100 × Bhima Shweta, MS 100 × W-448, Bhima Kiran, Bhima Shubhra, Bhima Shweta
Cluster II	26	Acc. 1608, Acc. 1609, Acc. 1613, Acc. 1622, Acc. 1635, Acc. 1649, Acc. 1656, Acc. 1657, Acc. 1658, Acc. 1663, W-009, W-085, W-340, W-396, W-397, W-414, W-419, W-439, W-441, W-444, W-448, W-453, DOGR 1044, DOGR 1048, RGP-2, N-2-4-1
Cluster III	36	Acc. 1612, Acc. 1617, Acc. 1621, Acc. 1623, Acc. 1625, Acc. 1632, Acc. 1633, Acc. 1636, Acc. 1637, Acc. 1652, Acc. 1660, Acc. 1664, Acc. 1669, Acc. 1694, Acc. 1700, W-132, W-344, W-355, DOGR 1050, DOGR 1172, DOGR Hybrid-1, DOGR Hybrid-2, DOGR Hybrid-5, DOGR Hybrid-6, DOGR Hybrid-7, DOGR Hybrid-50, DOGR Hybrid-441, KH-M-1, KH-M-2, MS 100 × W-361, RGP-3, RGP-5, P-1-3, Bhima Red, Bhima Safed, Bhima Super
Cluster IV	13	Acc. 1615, Acc. 1627, Acc. 1629, Acc. 1639, Acc. 1651, Acc. 1655, Acc. 1661, Acc. 1668, KH-M-3, KH-M-4, Bhima Light Red, Bhima Raj, Phule Safed
Cluster V	9	Acc. 1619, Acc. 1646, W-306, W-504, W-172, RGP-1, RGP-4, Bhima Dark Red, Bhima Shakti

#### Highly Tolerant Genotypes (Cluster II Group)

Twenty-six genotypes accounting for 26% of the total genotypes were placed in this group. These genotypes performed superiorly under water deficit stress with improved physiological and biochemical traits, especially higher plant water status, total chlorophyll, MSI, antioxidant enzyme activity, and photosynthetically active leaves that contributed to its higher bulb yield. Hence, these genotypes were identified as highly drought-tolerant genotypes (Table 6). Seven highly drought-tolerant genotypes of this group (Acc. 1656, Acc. 1658, W-009, W-448, W-444, W-397, and W-085) recorded less than 20% bulb yield reduction under drought stress (Table 1). These genotypes recorded considerable genetic diversity from the other tolerant genotypes as shown by the Euclidean distance in a dendrogram. Thus, these genotypes can be utilized in a breeding program to genetically improve this valuable crop for drought tolerance.

#### Tolerant Genotypes (Cluster I Group)

Sixteen genotypes were placed in this group, accounting for 16% of the total genotypes. These genotypes performed better under drought stress with improved physiological and biochemical traits and minimum bulb yield reduction (Table 1). The mean values for important traits, namely DTE (59.32), SSI (0.70), yield reduction (40.68), MSI (72.15), RWC (65.12), total chlorophyll (5.68), and leaf area (35.76) (Table 6). Therefore, these genotypes were identified as drought-tolerant genotypes with better phenotypic and biochemical traits.

#### Intermediate Genotypes (Cluster III Group)

Thirty-six genotypes were placed in this cluster group forming 36% of the total genotypes. These genotypes are intermediate in their drought tolerance and sensitive in their performance for all the studied physiological and biochemical parameters. The mean values for important physiological traits, namely DTE (39.00), SSI (1.64), yield reduction (61.00), MSI (72.17), and RWC (65.20) (Table 6). Thus, these genotypes were identified as intermediate genotypes that performed according to the external environmental condition, particularly the water regimes.

#### Highly Sensitive Genotypes (Cluster IV Group)

Thirteen genotypes were placed in this cluster, accounting for 13% of the total genotypes. These genotypes were recorded with poor DTE and with the least performance for physiological and biochemical traits. Among these genotypes, the two most sensitive were Acc. 1627 and Acc. 1639. The mean values for crucial traits, namely DTE (20.27), SSI (4.37), yield reduction (79.73), MSI (66.22), RWC (56.81), total chlorophyll (2.70), and leaf area (32.14) (Table 6). Thus, these genotypes were categorized as highly sensitive genotypes.

#### Sensitive Genotypes (Cluster V Group)

Nine genotypes were placed in this cluster, accounting for 9% of the total genotypes. These genotypes were characterized as sensitive with low DTE and poor performance for physiological and biochemical traits. The mean values for important traits, namely DTE (32.58), SSI (2.13), yield reduction (67.42), MSI (65.95), RWC (57.70), total chlorophyll (3.03), and leaf area (31.44) (Table 6). These genotypes performed well with optimum irrigation and could recover from the water stress after subsequent irrigation. However, they failed to form good size bulbs after recovery and hence were classified as drought-sensitive genotypes.

### Principal Component Analysis

**FIGURE 2:**
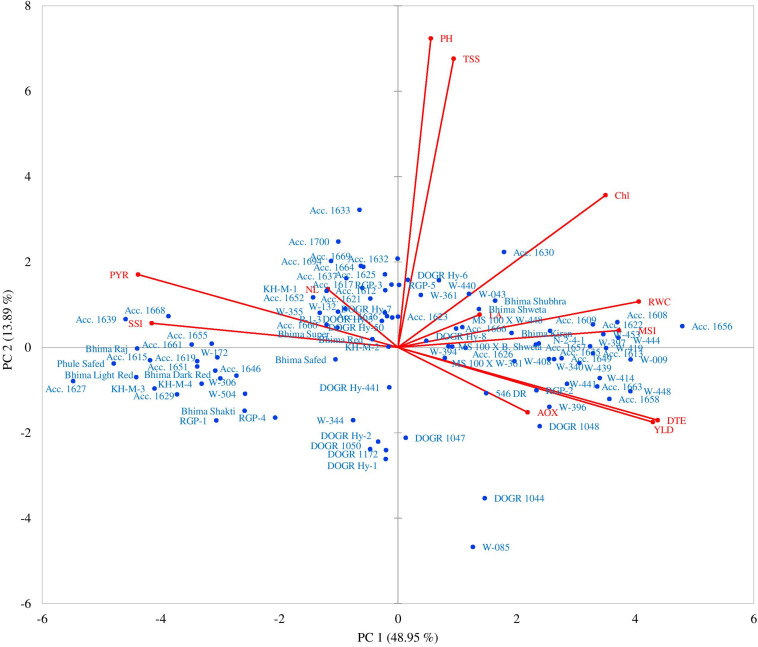
Principal component biplots presenting grouping of 100 onion genotypes and distribution of different traits under drought stress.

The rotated component matrix presented in Table 10 shows the percentage of total variance elucidated by different principal component groups and their correlation with the studied traits. The PCA resulted in three principal component groups having an eigenvalue of more than one, thus contributing 73.64% variability (Figure 2). The PC1, PC2, and PC3 groups contributed 48.94, 13.89, and 10.80% variability, respectively. Different parameters contributed both positively and negatively to different PC groups. In the PC1 group, DTE (0.390), yield (0.383), RWC (0.362), MSI (0.332), and chlorophyll (0.312) recorded the highest variability. By contrast, SSI, percent yield reduction, and the number of leaves contributed negatively to the PC1 group (−0.370, −0.390, and −0.105, respectively). Maximum variability was observed for plant height (0.645), TSS (0.602), and chlorophyll (0.317) in the PC2 group, whereas the number of leaves (0.656) recorded higher variability than the other traits in the PC3 group. Thus, PC1 group showed the highest variability (48.94%) for drought tolerance-contributing traits than the other groups.

**TABLE 10:** Rotated component matrix for the principal components of 12 traits of 100 onion genotypes evaluated under drought stress.

Parameters	PC-1	PC-2	PC-3
DTE	**0.390**	–0.152	–0.118
SSI	–0.370	0.050	–0.032
YL%	–0.390	0.152	0.118
TSS	0.084	**0.602**	–0.171
AOX	0.195	–0.136	**0.464**
MSI	**0.332**	0.035	**0.246**
RWC	**0.362**	0.096	0.071
CHL	**0.312**	**0.317**	–0.137
LA	0.123	0.068	**0.440**
PH	0.050	**0.645**	0.047
NL	–0.105	0.121	**0.656**
YLD	**0.383**	–0.156	–0.124
Eigenvalue	**5.874**	**1.667**	**1.297**
Variability (%)	**48.947**	**13.891**	**10.808**
Cumulative variance%	**48.947**	**62.838**	**73.645**

*DTE, drought tolerance efficiency; SSI, stress susceptibility index; YL%, yield loss percentage; TSS, total soluble solids; AOX, antioxidant enzyme activity; MSI, membrane stability index; RWC, relative water content; CHL, chlorophyll; LA, leaf area; PH, plant height; NL, leaves plant^–1^; YLD, bulb yield; PC, principal component. Values in bold indicating maximum variability.*

The PCA further revealed the association between different parameters and genotypes as demonstrated by the principal component biplots (Figure 2) for drought stress. Genotypes × parameter biplots were created from a two-way matrix of 12 parameters and 100 onion genotypes for water-deficit stress conditions. A smaller angle between different parameters in the same direction indicated a high association between corresponding parameters for classifying genotypes. Genotypes superior for a particular parameter were plotted closer and along the vector line direction. Under the water deficit condition, the genotypes were mostly concentrated on the positive side of both, PC1 and PC2. The genotypes Acc. 1656, Acc. 1658, Acc. 1608, Acc. 1622, Acc. 1663, W-009, W-444, W-397, W-396, W-414, W-448, W-441, and RGP-2 were more inclined in the direction of bulb yield, DTE, RWC, MSI, leaf area, and antioxidant enzyme activity. The highly sensitive genotypes were categorized based on cluster analysis; Acc. 1639, Acc. 1627, Acc. 1629, Acc. 1615, Acc. 1668, Acc. 1651, Acc.1655, KH-M-3, KH-M-4, Bhima Raj, Bhima Light Red, and Phule Safed were clustered together in the direction of SSI and percent yield reduction. These genotypes showed inclination on the negative side ofPC1. The PCA thus confirmed that genotypes excelling in drought-adaptive traits namely RWC, MSI, total chlorophyll, antioxidant enzyme activity, and DTE, contributed maximum toward bulb yield under water stress than the other traits. Taken together, genotypes performing superior for these traits can thus be identified as drought-tolerant genotypes.

## Discussion

Drought stress differentially affected morpho-physiological, and yield attributes of the onion crop depending on the plant growth stage and genotype sensitivity (Pelter et al., 2004). Onion breeders mainly aim at screening effective germplasms by using phenotypic and drought indices for developing drought-tolerant varieties (Ghodke et al., 2020). To date, information regarding the screening of onion genotypes under drought stress is limited. To address this issue, 100 onion genotypes were screened on the basis of drought indices, and physiological, biochemical, and yield traits. In our study, drought stress severely altered the crop physiology and biochemical mechanisms, ultimately reducing the bulb yield in all the tested genotypes. The challenge was more pronounced during the bulb development stage. Based on DTE and percent yield loss the genotypes were classified as highly tolerant, tolerant, intermediate, sensitive, and highly sensitive. Genotypes with higher DTE had lower bulb yield reduction under drought stress than the other genotypes. The present study results are consistent with those of previous study, where 35 wheat genotypes were screened on the basis of DTE and tolerant genotypes with high DTE were identified (Ghodke et al., 2019). This stress indices might help the tolerant genotypes to adjust and sustain under stressful environments. Likewise, highly significant genotypic differences were observed for all the studied physiological, biochemical, and yield traits under drought stress, indicating that the tested genotypes had inherent genetic differences among themselves for the studied traits. Genotypes × environment interactions were also significant for various traits indicating that most of these traits were quantitatively inherited and differentially expressed in response to a diverse environment. A recent study in wheat also reported supportive findings for genotypes, treatments, replications, and their interaction effects (Mwadzingeni et al., 2016; Arifuzzaman et al., 2020). The findings thus revealed the huge genetic diversity exhibited by the studied genotypes that can be exploited in breeding programs.

### Effect of Genotypes and Drought Stress on Physiological Traits

Drought stress negatively influenced the plant phenotype by decreasing plant height, photosynthetically active leaves, and leaf area in all the employed genotypes. Plant height may be reduced due to a decline in cell growth and expansion that limits the overall plant architecture under drought stress. Similar changes in plant height, leaf area, and root growth were observed during the vegetative and reproductive phases of soybean crops under drought stress (Yan et al., 2020). Our previous findings in the onion crop support the present results, that drought stress severely affects plant height, leading to stunted growth (Ghodke et al., 2018). Further, drought stress significantly affects the photosynthesis apparatus and pigments that decrease the photosynthesis rate, light-harvesting mechanism, and assimilate partitioning in plants (Cornic and Massacci, 1996). Leaf senescence is a typical symptom under drought stress that increases with stress severity. Genotypes with higher chlorophyll content maintained their optimum photosynthesis rate to meet the crop requirement under limited water supply. Reduction in the number of leaves, and chlorophyll pigments, and a higher leaf senescence rate with a declined leaf area were found in the tested genotypes under water-deficit stress compared with well-watered conditions. These alterations in traits by defoliating functional leaves and reducing chlorophyll pigments adversely affect the overall photosynthesis process. Differential behavior was observed among the genotypes for these traits possibly due to their inherent divergence and tolerance mechanism under stress. Selective genotypes, namely Acc. 1656, Acc. 1658, W-009, W-448, and W-397, maintained their leaf area and chlorophyll pigments under both water regimes and were therefore categorized as drought tolerant. By contrast, few genotypes viz. Acc. 1627 and Acc. 1639, that exhibited a high leaf senescence rate and low chlorophyll with poor photosynthesis were designated as drought-sensitive. Several studies in other crops, such as wheat (Mwadzingeni et al., 2016), green gram (Baroowa et al., 2016), and black gram (Gurumurthy et al., 2019), have reported similar findings. In our study, genotypic variation in RWC was observed under drought stress in the tested genotypes. The tolerant genotypes with high RWC maintained cellular turgidity, which might have helped them to survive under stress through a desiccation tolerance mechanism. MSI is another important drought adaptive trait that is mostly over-expressed under the water-deficit stress condition compared with the well-watered condition. This trait is important in overcoming cellular injury or membrane damage caused by free radicals under water stress. The increasing MSI level was reported in most tolerant genotypes compared with the sensitive ones. The increase in MSI can directly be used for assessing stress-induced injury in numerous genotypes. Plants develop a complex defense mechanism (Enzymatic and Non-enzymatic antioxidant activity) to cope with oxidative stress induced by reactive oxygen species (Sairam et al., 1997). Antioxidant enzyme activity increases sharply under drought stress in crop such as leafy vegetables (Sarker and Oba, 2018), tomato (Aghaie et al., 2018), and muskmelon (Ansari et al., 2017). All these previous reports have revealed that genotypes with high antioxidant enzyme activity perform better under drought stress. Phenolic compounds generally increases during water stress (Hodaei et al., 2018). In the tolerant genotypes levels of total phenol recorded increased as compaired to well-watered In our study, considerable genotypic variation was observed for these adaptive traits particularly, RWC, MSI, and antioxidant enzyme activity. Tolerant genotypes (Acc. 1656, Acc. 1658, W-009, and W-085) performed superiorly under drought stress by excelling in these traits as the stress increases. By contrast, sensitive genotypes (Acc. 1627, Acc. 1639, Bhima Raj, Bhima Light Red, and Phule Safed) with high SSI and poor yield responded negatively for these traits under drought stress. Our recent findings also support the results of the present study, where a drought-tolerant onion genotype had higher RWC, MSI, and antioxidant enzyme activity than the sensitive genotype (Ghodke et al., 2020). The findings were further supported by the study of Chaturvedi et al. (2019) in okra, where plant water status and cellular membrane stability decreased under drought stress during the crop vegetative and reproductive stage. The phenols are non-enzymatic antioxidants, and there accumulation reported in biotic and abiotic stress (Hodaei et al., 2018). Together, these traits can be believed to play an adaptive role in drought tolerance and can be used as a selection criterion for screening large germplasm pool for tolerant genotypes.

In our study, the value for the PCV was higher than that for the GCV for all the studied traits among the tested genotypes, signifying the influence of external environmental factors on various plant traits. The low heritability value further indicates the less genotypic variance among the tested genotypes. The higher influence of water deficit stress on the expression of adaptive traits might help in screening genotypes under the water stress environment.

### Effect of Drought Stress on Yield and Its Contributing Traits

Previous studies have shown that drought stress significantly reduces onion bulb yield (Ghodke et al., 2018; Wakchaure et al., 2018). Variation was observed in the present study for bulb yield and its associated traits due to genetic divergence among the genotypes under the well-water and water-deficit stress conditions. The results of the pooled analysis for 2 years, revealed genotypes with optimum bulb yield under both water regimes with minimum yield reduction under water stress; these genotypes can be further employed in breeding programs. A significant yield difference was observed among the genotypes under the well-watered and water-deficit conditions, with most sensitive genotypes being severely affected by drought stress during the bulb development stage. Bulb yield reduction might be due to drought-induced reduction in leaf photosynthesis and the translocation process toward developing bulbs. Consequently, bulbs of small size and poor marketable quality were produced. Thus, unique tolerant genotypes with improved drought-adaptive traits can be useful for achieving the potential yield in different drought-prone areas. The results of various studies on different crops, such as maize, wheat (Webber et al., 2018), beans (Darkwa et al., 2016), and potato (Banik et al., 2016), where drought stress-induced yield losses are observed, support our findings.

### Association Among Different Traits Under Drought Stress

The correlation analysis showed a significantly higher positive correlation between DTE and GMP and a negative association between DTE and SSI, indicating that genotypes with high DTE and low SSI have less bulb yield difference under the two water regimes. A strong positive association was also observed between bulb yield and physiological traits such as RWC, MSI, antioxidant enzyme activity, and total chlorophyll content under drought stress. Thus genotypes that maintained its water status, prevented membrane damage by enhancing antioxidant enzyme activity, and regulate its photosynthesis activity under limited water supply had a better drought tolerance mechanism than the other genotypes. These findings thus revealed the prominence of these traits and drought indices in selecting tolerant genotypes for drought stress. The present findings are supported by previous study results in wheat (Golabadi et al., 2006; Mohammadi, 2016; Ghodke et al., 2019) and safflower crop (Bahrami et al., 2014) under the drought environment.

Multivariate analysis further revealed the relationship between more than two traits at the same time through cluster analysis, descriptive statistics, and PCA. Cluster analysis produced a broad range of variability that helped in identifying tolerant genotypes. The cluster dendrogram was created using a Euclidean distance of 100 onion genotypes for drought conditions that grouped the genotypes into five clusters. Cluster II contained genotypes that exhibited more drought tolerance and less stress susceptibility with improved physiological and biochemical traits such as higher RWC, MSI, total chlorophyll, antioxidant enzyme activity, and leaf area. These genotypes were designated as highly drought-tolerant. Again, cluster I contained the tolerant genotypes that performed better for in terms of various physiochemical traits under limited water supply. Furthermore, genotypes with an intermediate performance for all physiological and yield traits were placed in cluster III. These genotypes, recorded good yield and growth behavior under the well-watered condition, whereas they failed to maintain their potential bulb yield under moderate to severe drought stress. The physiological traits, viz. RWC, MSI, and total chlorophyll, were severely affected due to water stress in genotypes of cluster IV. Poor bulb yield in genotypes of this group under water stress may be due to increased membrane damage, low plant water status, and altered enzyme activities which increased their sensitivity to drought. Likewise, genotypes of cluster V were categorized as sensitive as they performed relatively under mild stress and substantial yield reduction was observed under severe stress. This method of grouping genotypes based on their phenotypic traits and yield performance has been followed for many crops. In tomato crop, 14 cultivars were grouped into four clusters, with tolerant cultivars placed in cluster I with a high yield potential and proline level, and sensitive and highly sensitive cultivars with a low proline level and severe membrane damage under moderate and severe drought stress were placed in cluster III and cluster IV, respectively (Aghaie et al., 2018). In our previous study, wheat genotypes were grouped into five clusters based on their drought tolerance and physiological performance under drought stress (Ghodke et al., 2019).

The PCA in the present study indicated that under drought stress, DTE, RWC, MSI, total chlorophyll, and antioxidant enzyme activity significantly influence bulb yield. Jan et al. (2018) showed that PC groups having an eigenvalue more than 1 exhibit greater variability than the PC group having an eigenvalue of less than 1, which supports our findings. According to this criterion, the first three PC groups contributed 73.64% of the total variability under drought stress, thereby clearly indicating the structure underlying the traits analyzed. In the PC1 group, maximum variability was observed for DTE, which indicates that this parameter can act as a promising trait for screening numerous onion genotypes under drought stress. Genotypes with high DTE performed superiorly with high yield and adaptive phenotypic responses under mild to severe drought stress. Arifuzzaman et al. (2020) showed that the maximum variability under drought stress was contributed by PC1 where leaf collar height of wheat genotypes accounted for the highest positive contribution, whereas the negative contribution of the chlorophyll content and root to shoot ratio was the highest. Similar findings were reported in a wheat crop, thus supporting our result for onion genotypes (Mwadzingeni et al., 2016).

A biplot created between PC1 and PC2 showed a clear pattern of grouping genotypes along the vector line. The outstanding performance of particular genotypes for a specific trait was plotted closer to the vector line. Genotypes and traits that lie far away from the origin have better breeding potential than the other genotypes. In this study, the genotypes Acc. 1656 and W-085 were at a considerable distance from the origin and hence can be utilized in onion breeding program as a tolerant genotype. Again, Acc. 1656 was close to the vector line and was associated with drought-tolerant characters (DTE, MSI, RWC, and antioxidant enzyme activity), and thus, it can be employed for developing a drought-tolerant onion variety. Similar findings in wheat were reported by Grzesiak et al. (2019) and Arifuzzaman et al. (2020) where drought-tolerant genotypes were plotted close to the drought adaptive promising traits and were found to be suitable for the wheat breeding program. These findings indicated that maintenance of high DTE contributes maximum (more acute angle) to bulb yield compared with other traits under drought stress. However, RWC, MSI, and antioxidant enzyme activity also contribute to bulb yield under drought stress by accelerating various adaptive physiological and biochemical mechanisms.

Overall, the findings suggested that drought indices, PCA, and hierarchical cluster analysis could be used as reliable methods for screening onion genotypes and classifying them into different categories based on the variation in phenotypic traits, and physiological, biochemical, and yield performance under drought stress. This approach can also be utilized for screening the onion germplasm for other biotic and abiotic stresses and for identifying the most contrasting genotypes for a particular stress. Here, the tolerant genotypes identified might carry genes for drought tolerance that may act as a useful tool in onion breeding program for drought tolerance. Further, these unique tolerant genotypes can be crossed with high yielding popular onion varieties to introgress the genes for drought tolerance in the variety without affecting its inherent yield potential under drought stress.

## Data Availability Statement

The original contributions presented in the study are included in the article/Supplementary Material, further inquiries can be directed to the corresponding author/s.

## Author Contributions

PG: project administration, resources, supervision, methodology, analysis, and writing original draft. AT: project administration, resources, supervision, and writing–original draft. DS: methodology, biochemical analysis, statistical analysis, writing–original draft, writing–review, and editing. SG: methodology, statistical analysis, and writing–original draft. KB: writing–original draft, and statistical analysis. OS: methodology, statistical analysis, writing–review, and editing. AG and VM: resources. PS: methodology and writing–original draft. VS and YK: methodology and analysis. SG: project administration, resources, supervision, writing–original draft, writing–review, and editing. PH and RR: methodology and statistical analysis. MS: project administration, resources, and supervision. All authors listed have made substential, direct and intellectual contribution to the work.

## Conflict of Interest

The authors declare that the research was conducted in the absence of any commercial or financial relationships that could be construed as a potential conflict of interest.

## Footnotes

1 http://agricoop.gov.in/

## Supplementary Material

Supplementary Figure 1 Monthly temperature and rainfall patterns recorded during the experiment period for two constitutive years (2017–2018 and 2018–2019).
