# Supplementary figures and images for: Screening of Onion (Allium cepa L.) Genotypes for Drought Tolerance Using Physiological and Yield Based Indices Through Multivariate Analysis
Source: Front Plant Sci. 2021 Feb 9;12:600371. doi: 10.3389/fpls.2021.600371 (PMC7900547; doi:10.3389/fpls.2021.600371)

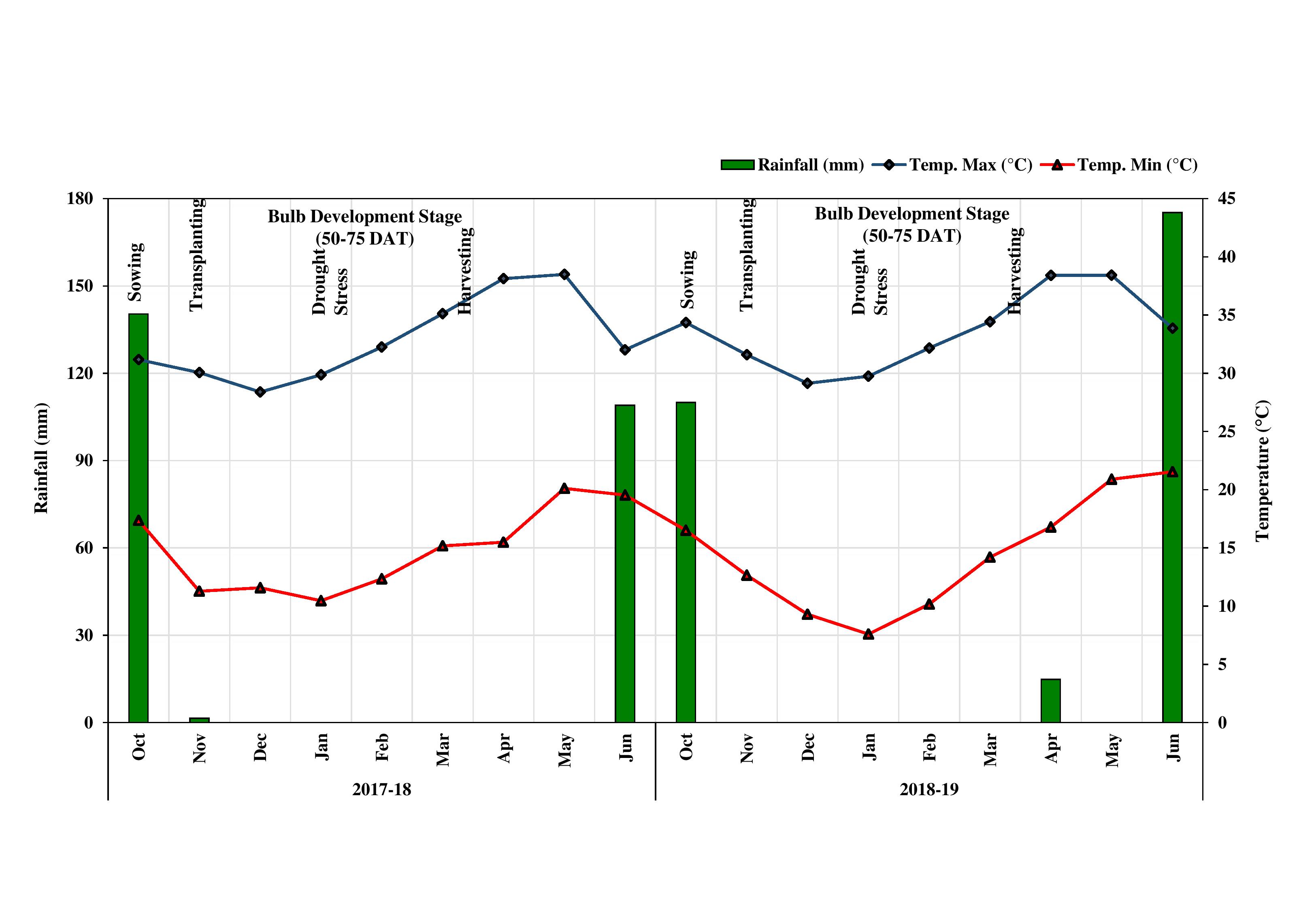

Supplement: Supplementary Figure 1 — Monthly temperature and rainfall patterns recorded during the experiment period for two constitutive years (2017–2018 and 2018–2019). [file Image_1.JPEG]
