# Supplementary material for: Screening of Onion (Allium cepa L.) Genotypes for Drought Tolerance Using Physiological and Yield Based Indices Through Multivariate Analysis
Source: Front Plant Sci. 2021 Feb 9;12:600371. doi: 10.3389/fpls.2021.600371 (PMC7900547; doi:10.3389/fpls.2021.600371)
Supplement: Supplementary file 2 [file Table_1.DOC]

**Climatic Conditions during Experimental Period**

The experiment was carried out for two years 2017-18 and 2018-19 during the post monsoon season. The amount and distribution of rainfall, minimum and maximum temperature, and sunshine hours differed greatly during the two planting period as given in Supplementary Figure 1. The influence of climatic conditions during two cropping seasons on bulb yield differed significantly among the genotypes. This indicates the significant effect of climatic variables on the performance of onion genotypes.

**Supplementary Figure 1.** Monthly temperature and rainfall patterns recorded during the experiment period for two constitutive years (2017-18 and 2018-19).
